# Engaging the University’s Retired Community

**DOI:** 10.1093/geroni/igaa057.1732

**Published:** 2020-12-16

**Authors:** Pamela Elfenbein

**Affiliations:** University of North Georgia, Gainesville, Georgia, United States

## Abstract

In support of our older adult community and in alignment with Age Friendly University principles, the University of North Georgia Center for Healthy Aging promotes wellness and disease prevention through active research and intervention, promotes leadership through mentorship and partnerships, fosters an environment of healthy aging through interdisciplinary service and research with community partners, and supports healthy aging through innovative interprofessional collaboration and research. This presentation offers insight into the ways we have engaged retired faculty and staff, with particular focus on a collaboration with The Wisdom Project, a community organization of adults 55+ interested in using their skills and talents to benefit their community through action and advocacy projects. While aligned with Principle 9, this presentation also offers insight regarding how the university supports additional AFU Principles.

